# Theoretical implementation of prior knowledge in the design of a multi-scale prosthesis satisfaction questionnaire

**DOI:** 10.1186/s12938-016-0288-5

**Published:** 2016-12-19

**Authors:** Tim Schürmann, Philipp Beckerle, Julia Preller, Joachim Vogt, Oliver Christ

**Affiliations:** 1 Work and Engineering Psychology, Department of Psychology, Technische Universität Darmstadt, Darmstadt, Germany; 2 Institute for Mechatronic Systems in Mechanical Engineering, Technische Universität Darmstadt, Darmstadt, Germany; 3 Institute Humans in Complex Systems, School of Psychology, University of applied Arts and Sciences Northwestern Switzerland, Olten, Switzerland

**Keywords:** Prosthetics, Lower limb amputation, Psychological adjustment, Bayesian inference

## Abstract

**Background:**

In product development for lower limb prosthetic devices, a set of special criteria needs to be met. Prosthetic devices have a direct impact on the rehabilitation process after an amputation with both perceived technological and psychological aspects playing an important role. However, available psychometric questionnaires fail to consider the important links between these two dimensions. In this article a probabilistic latent trait model is proposed with seven technical and psychological factors which measure satisfaction with the prosthesis. The results of a first study are used to determine the basic parameters of the statistical model. These distributions represent hypotheses about factor loadings between manifest items and latent factors of the proposed psychometric questionnaire.

**Methods:**

A study was conducted and analyzed to form hypotheses for the prior distributions of the questionnaire’s measurement model. An expert agreement study conducted on 22 experts was used to determine the prior distribution of item-factor loadings in the model.

**Results:**

Model parameters that had to be specified as part of the measurement model were informed prior distributions on the item-factor loadings. For the current 70 items in the questionnaire, each factor loading was set to represent the certainty with which experts had assigned the items to their respective factors. Considering only the measurement model and not the structural model of the questionnaire, 70 out of 217 informed prior distributions on parameters were set.

**Conclusion:**

The use of preliminary studies to set prior distributions in latent trait models, while being a relatively new approach in psychological research, provides helpful information towards the design of a seven factor questionnaire that means to identify relations between technical and psychological factors in prosthetic product design and rehabilitation medicine.

## Background

Traditional product development uses several established methods and tools to organize its development phases and implement design aspects relevant. In rehabilitational product design like prosthetics, it is crucial to consider the needs of the users because they are literally connected to their prosthesis and will be accordingly affected by bad design decisions.

Able-bodied persons can complete tasks normally with the help of their limbs and sensorimotor body functions and the loss of body parts through amputation can cause severe physiological and psychological trauma [1]. In a rehabilitation context, psychological phenomena such as phantom limb pain or the rubber hand/foot illusion [2] show that adapting to a product which replaces a lost body part is a challenge to the body’s sensorimotor integrity. Therefore, approaches to include the user in the development of prosthetics by using methods like the quality function deployment [3] or concept simulators [4] have been proposed [5, 6].

### Current state of prosthetic satisfaction research

How do currently available questionnaires deal with the difficulties mentioned above? The most commonly used questionnaires in prosthetics deal with impairments on the user’s quality of life and the psychosocial adjustments to life with a prosthesis [7, 8] as well as their impact on product satisfaction. They partially explore the presence of phantom limb pain and processes involving the body image [9] but their scales are not well suited to provide useful information for product development or the “human in the loop” approach. User satisfaction with the prosthesis if measured on one dimension, shows a positive correlation between product satisfaction and activity restrictions [10] which indicate an effect on satisfaction caused by multiple independent sources. This article proposes a questionnaire based on the latent trait model described in [11–13] aimed at prosthesis users. It shares similarities to other theoretical sources of amputee needs [9] but differs with regard to the implementation and interaction of technical and psychological factors.

### Probabilistic inference

To facilitate understanding of the methodology of this paper, this section provides a short introduction to probabilistic inference. As opposed to frequentist inference, probabilistic inference involves the formulation and testing of hypotheses by establishing probability distributions of the values the variables and parameters in question can take on. This process begins with the formulation of a prior distribution. In a given area of research, an effect variable A has on variable B might be common knowledge because it has been found and replicated in a number of studies. Sometimes the found effect is smaller or larger than before but the distribution of these previously found effects can and should be integrated into the current analysis. Instead of assuming no knowledge about the effect under investigation when we start gathering data we can establish the prior distribution as probabilistic information about the size of the effect parameter. Given what we know up until this point in time we then start gathering and interpreting data, arriving at another distribution of values. In probabilistic terms, the parameter distribution of our data is called the likelihood. Bayes’ theorem offers the mathematically appropriate way to combine both distributions to arrive at a posterior estimate. Given both our prior knowledge about the proposed effect and the new data, the posterior distribution is the best estimate of the effect we are interested in investigating. Besides its purely data-analytical application current research provides evidence that some form of probabilistic inference underlies human perception and decision making. This is even true up to a point where sensorimotor events can best be described by using Bayesian inference [14]. In a scenario like in this paper where a complex statistical analysis takes place and there are many parameters to determine, Bayesian inference has a different set of requirements than frequentist inference would impose on the data. In addition, it makes obtaining a credible estimate of parameter values inside a statistical model relatively straightforward. Especially the notion and common confusion about confidence intervals becomes an issue in this study: If one is interested in designing a reliable measurement tool such as the proposed questionnaire, one needs to understand the probability of one item influencing one factor with relative certainty or learn about the most likely values of a regression parameter between one factor and another. In this scenario a regression parameter between factors describes how much the value of one factor increases or decreases depending on the value of another one. Frequentist analysis does not provide that information in an easily interpretable manner. “Highest Credibility Intervals” or “Highest Density Intervals” (HDI) [15] on the other hand do. In this paper, we utilize prior knowledge about parameters of the model by means of an expert study. But even experts cannot judge with absolute certainty, just like we cannot be completely certain about how large the value of a factor loading really is. This uncertainty is reflected in the width of an HDI, making the spectrum of probable values extremely large or relatively narrow. By gaining prior information about the parameter values of the proposed latent trait model, we try to state hypotheses about the psychometric evaluation of this questionnaire more precisely than before, making inferences drawn from data more reliable.

### Psychometric evaluation via Bayesian inference

In order to psychometrically validate the latent trait model, two methodological steps are followed: firstly, the items are each assigned to one factor and the resulting model is tested by means of confirmatory factor analysis. Secondly, for each factor formed out of a linear combination of its corresponding items, the relationships between factors is assessed via linear regression.

By applying Bayesian inference methodology, one is able to assign prior distributions to each of the assessed parameters described above. Prior knowledge about each item’s factor loading as well as the regression coefficients between factors can be described by a prior distribution, implying hypotheses about the quantitative nature of the parameter. When there is a lack of prior information, one uses what is known as an uninformative prior distribution, characterized by a centered mean and high standard deviation in a distribution. There is an ongoing debate in statistical science about whether the use of informed priors is valid in scientific research, but just as in regular hypothesis testing, it is favorable to include every available source of prior information [16] into a statistical model. In order to assess the legitimacy of the chosen prior distributions, one can cross-validate the statistical model by comparing it to a completely uninformed alternative to see if one’s choice of prior distributions affected the outcome of the analysis [15].

## Methods and expert evaluation

An expert study was included into the process of informing prior distributions in the latent trait model. It was conducted at the Technische Universität Darmstadt, Germany, in 2013.

### Assessment of expert agreement on item-factor classification

This first study assessed the level of agreement reached by 22 experts in the field of prosthetics when assigning each item (85 at the time) to a factor of the framework described [11–13] in a web-based sorting game. The experts did not answer the items on a scale as a prosthesis user would but rather had to assign them to the latent factor they felt the items belonged to the most. The factors in question were satisfaction (SAT), feeling of security (FoS), body schema integration (BSI), support (SUP), socket (SOC), mobility (MOB) and outer appearance (OUT), in addition to one rejection factor in case the expert felt like the item should be excluded from the item pool. The main result indicated a fair amount of agreement (AC1 index = 0.39) [17], with some factors rated noticeably more consistently than others. A measure of shared ratings was calculated which described the amount of times an item had been rated to belong to another factor than the one the majority of raters had assigned it to. The factors body schema integration, socket and outer appearance were rated quite distinguishably, with average shared ratings (ASR) of 4.97, 6.83 and 3.87% with other factors. On the other end of the shared rating measure, the factor Support showed the highest ASR with 19.87%. It also showed a very high shared rating with Mobility, which itself had an ASR of 10.86 percent. Feeling of security resulted in 11.32% ASR. While these results so far indicated some clearly distinguishable and some more difficult factors, the factor Satisfaction (13.99% ASR) showed a fairly high level of shared ratings with every other factor except for feeling of security. This measure serves purely as an account of how distinctive experts perceived the items of each factor to be with regards to other factors. The measure does not inform any model parameters in the future confirmatory factor analysis but it might correlate with a factor’s variance explained by its contributing items. 15 items were excluded from the item pool because the experts’ ratings were too diverse which made a clear factor classification impossible or because the items were rejected by a majority of experts. Raw data of this study (i.e. the frequency with which experts rated one item on different factors) were taken into account to form prior distributions about the item-factor classifications and their respective factor loadings.

### Transition from study results to prior distributions

The expert study provides information about how the measurement model underlying our questionnaire should be constructed. Item-factor classification is achieved via majority agreement in the preliminary study and forms the basis of the confirmatory factor analysis. Factor loadings between the manifest items and their corresponding latent factors are informed as follows. The preliminary study contains information about the factor loadings: an item X is assigned to a factor Y by the majority of experts which is assumed to load positively on that factor. The mean for a prior distribution of a factor loading will be set to the percentage of expert agreement on the item to its factor. For example, if an item has been assigned to its majority factor by 17 out of 22 experts, the mean of its factor loading distribution will be M = 0.77. The distribution’s standard deviation will be set to (1 − M)/2, in this case (1 – 0.77)/2, SD = 0.115. This way, whether an item was rated more unanimously than others will be shown in its prior factor loading and the uncertainty about the prior. All other coefficients, like measurement errors from items to their respective factors, will be implemented using uninformative priors.

## Results

### Item-factor classifications

The item-factor classifications were implemented through majority agreement in the preliminary study. For example, an item with 14 assignments towards satisfaction and less assignments to every other factor would be designated a satisfaction item for the confirmatory factor analytic setup. Items that did not achieve single majority or those which were predominantly rejected by the experts were excluded from the item pool. A list of item-factor classifications can be found in Table 1, with Table 2 providing a preliminary translation of the German items into English. The translations are suggestions which will be cross-validated in the future. In the final questionnaire assessment with prosthetic users, items will be measured on a 5-point Likert scale ranging from “No agreement” to “Complete Agreement” with an option to omit answering in order to respect a possible subject sensitivity towards specific items.

**Table 1:** Model factors and assigned items

Factor	Array of items
SAT	1, 3, 4, 5, 31, 48, 49, 50, 55, 69, 70
FoS	6, 7, 26, 27, 28, 40, 42, 47, 52, 64, 66
BSI	30, 56, 57, 58, 59, 60, 61, 62, 63, 65, 67
SUP	23, 24, 32, 33, 54
SOC	2, 8, 13, 14, 15, 16, 17, 18, 19, 20, 21, 22, 25, 68
MOB	12, 34, 35, 36, 37, 38, 39, 41, 43, 44, 45, 46, 51, 53
OUT	9, 10, 11, 29

**Table 2:** Preliminary translation of German questionnaire items into English

Item	Statement
1	In everyday life I am satisfied with the fit of my prosthesis
2	I am satisfied with the fit of my prosthesis to my stump
3	I am satisfied with the ability to walk when using my prosthesis
4	I am satisfied with the ability to sit when using my prosthesis
5	I am satisfied with the ability to walk when using my prosthesis
6	Keeping my balance while standing is easy for me
7	Keeping my balance while walking is easy for me
8	Putting on my prosthesis is easy for me
9	My prosthesis makes sounds (squeaking, clicking) frequently
10	I feel uncomfortable because of the sounds my prosthesis makes
11	I feel socially hindered because of the outer appearance of my prosthesis
12	I would consider a longer battery life of my prosthesis useful
13	I often experience pressure marks on my stump
14	I often experience swellings on my stump
15	After prolonged periods of wearing my prosthesis I experience problems with its fit
16	My prosthesis’ fit is regularly influenced by sweat inside it
17	I use a technical aid to improve the fit of my stump in the prosthesis
18	I have to adjust the socket of my prosthesis to my stump several times a day using additives
19	I use textiles (sock, liner) to adjust for the reduced volume of my stump
20	I use a technical aid to counteract the loss of volume of my stump
21	I regularly experience difficulties in everyday life due to a swollen stump
22	My prosthesis’ socket adjusts itself independently to the shape and volume of my stump
23	In walking, moving the prosthesis requires more muscle force than moving my unaffected leg
24	I would like my prosthesis to offer more support in order to reduce the amount of strength required for walking
25	I have pain in my phantom limb because of the prosthesis socket
26	I feel safe and stable during the stance phase of my prosthesis
27	I feel safe and stable during the swing phase of my prosthesis
28	I would like to have more stability during swing phase
29	I feel comfortable with my appearance when I’m in public
30	I feel like I have a natural gait
31	I am satisfied with my ability to stretch out the prosthesis
32	I would like my prosthesis to offer more mechanical/electronic support while extending it
33	I would like my prosthesis to offer more mechanical/electronic support while flexing it
34	I frequently adjust my speed while walking
35	I feel uncomfortable when I cannot change my speed while walking
36	I experience problems while trying to change my walking speed with my current prosthesis
37	I want to be able to change my walking speed on my own
38	I am satisfied with switching from standing to walking
39	Climbing stairs is difficult for me
40	I feel insecure about my prosthesis
41	My prosthesis is designed to climb stairs with it
42	I regularly stumble while climbing stairs
43	I can climb stairs by alternating between both feet
44	I climb stairs by placing the healthy leg on them first and following with the prosthesis
45	I can bend over with my prosthesis
46	I can lift heavy objects from the floor
47	I feel safe and secure while compensating for a loss of balance with my prosthesis
48	I am satisfied with suspension in initial heel contact
49	I am satisfied with suspension during extension
50	I am satisfied with suspension of my foot prosthesis
51	I have difficulty moving with my foot prosthesis while walking on uneven ground or up an incline
52	I regularly get caught on bumps with my foot prosthesis
53	I lift my foot by using my hip to get enough space to the ground
54	My prosthesis offers mechanical/electronic support while rolling over from heel to toes
55	I can adjust my prosthesis adequately regarding the heels of my shoes
56	When I look at my legs in everyday life, my prosthesis seems so belong to my body
57	The perception of my prosthesis in daily use is very close to the perception of the corresponding part of the body
58	I experience the prosthesis as a part of me
59	I experience the prosthesis as my new leg
60	I experience the prosthesis as part of my body
61	I perceive the prosthesis to be where I would expect the corresponding part of my body
62	I experience the prosthesis in the place of my former real part of the body
63	I feel the spatial position of my prosthesis without looking at it
64	I can feel ground properties with my prosthesis
65	I experience something touching my prosthesis as touching on the corresponding part of my body
66	It seems to me like I can control the prosthesis at all times
67	I can ease itching at the corresponding part of my body by scratching my prosthesis
68	I often have blisters on my stump because of an ill-fitting socket
69	I feel uncomfortable with the weight of my prosthesis in everyday use
70	I feel uncomfortable with the size of my prosthesis in everyday use

### Item-factor classifications

The means and standard deviations of prior distributions describing factor loadings will be set by taking expert agreement into account as described in the Methods section. Table 3 shows the complete list of Gaussian prior distributions of factor loadings. One exception with regards to this technique is item 47. It originally was assigned to the factor feeling of security by all the experts, creating a point estimate prior of M = 1, SD = 0. These values provide an unsuitable setup for a factor analysis. Factor loadings of exactly 1 are illogical because they imply a direct representation of the latent variable by one manifest variable. If such a variable existed, the whole process of creating the latent variable would be redundant. Therefore, we choose to provide item 47 with a distribution resembling the mean prior of other items on the factor feeling of security, equaling M = 0.72 and SD = 0.15. Prior distributions on the intercepts in the linear regression of factor loadings will be left uninformed. Since measurement errors are another type of parameter which are hard to estimate a priori, they will be handled as proposed by [18] by using inverse Gamma distributions (0.01, 0.01).

**Table 3:** Items and their individual factor loading prior distributions (FLPD) with M(SD)

Item	FLPD	Item	FLPD	Item	FLPD	Item	FLPD	Item	FLPD
1	0.91 (0.05)	15	0.77 (0.12)	29	0.82 (0.09)	43	0.59 (0.21)	57	0.91 (0.05)
2	0.64 (0.18)	16	0.82 (0.09)	30	0.50 (0.25)	44	0.77 (0.12)	58	0.82 (0.09)
3	0.68 (0.16)	17	0.59 (0.21)	31	0.55 (0.23)	45	0.50 (0.25)	59	0.86 (0.07)
4	0.59 (0.21)	18	0.77 (0.12)	32	0.45 (0.28)	46	0.73 (0.14)	60	0.86 (0.07)
5	0.73 (0.14)	19	0.68 (0.16)	33	0.45 (0.28)	47	0.72 (0.15)	61	0.86 (0.07)
6	0.68 (0.16)	20	0.64 (0.18)	34	0.77 (0.12)	48	0.55 (0.23)	62	0.73 (0.14)
7	0.86 (0.07)	21	0.77 (0.12)	35	0.68 (0.16)	49	0.68 (0.16)	63	0.64 (0.18)
8	0.45 (0.28)	22	0.86 (0.07)	36	0.68 (0.16)	50	0.59 (0.21)	64	0.55 (0.23)
9	0.77 (0.12)	23	0.45 (0.28)	37	0.77 (0.12)	51	0.55 (0.23)	65	0.91 (0.05)
10	0.68 (0.16)	24	0.59 (0.21)	38	0.41 (0.30)	52	0.55 (0.23)	66	0.59 (0.21)
11	0.91 (0.05)	25	0.77 (0.12)	39	0.73 (0.14)	53	0.59 (0.21)	67	0.59 (0.21)
12	0.45 (0.28)	26	0.91 (0.05)	40	0.86 (0.08)	54	0.55 (0.23)	68	0.82 (0.09)
13	0.95 (0.03)	27	0.82 (0.09)	41	0.55 (0.23)	55	0.45 (0.28)	69	0.41 (0.30)
14	0.91 (0.05)	28	0.73 (0.14)	42	0.59 (0.21)	56	0.77 (0.12)	70	0.64 (0.18)

### Latent variable values

The values of the individual latent variables (the seven factors in the model) will be set to represent uninformative Gaussian priors (M = 0, SD = 100).

### Summary of prior information derived from the expert study

In total, a measurement model of 70 manifest items constituting 7 latent variables with the described connections between factors results in 217 parameters. The preliminary study established an expert rating to determine which item would be assigned to which factor. The quantitative nature of their factor loadings was derived from the distribution of expert votes on each item. Items that were more unanimously ranked obtained a prior distribution of their factor loadings with higher absolute values than items that were rated more diversely (70 informed parameters). Structural Equation Modeling also requires some types of parameters that are hard to judge a priori via preliminary studies. These include regression intercept parameters and measurement error variances between items and factors (140 parameters) and the resulting values of a participant on each factor (7 parameters). As described previously, these 147 parameters will be set to resemble uninformed prior distributions.

## Conclusion

### Using prior knowledge to inform parameter estimation in complex models

The methodology of this paper involved using prior knowledge gathered from a preliminary study to inform parameter estimation for the latent trait model of the prosthesis satisfaction questionnaire. To do so and use a probabilistic instead of a frequentist approach grants the advantage of not having to deal with many of the limitations and requirements of regular structural equation modeling. Estimations of required sample sizes using frequentist methodology, for example, would quickly rise up to circa 500 subjects or more, depending on the number of parameters the researcher wishes to include. Especially when dealing with specific target groups, this can turn out to be a problem. Using probabilistic methodology, low sample sizes do not imply problems like this while high sample sizes do not guarantee a statistically significant result, as is the issue with traditional p values. Every participant of the upcoming questionnaire will merely reduce uncertainty in the parameter estimation without changing its outcome thereby decreasing the risk of creating a false alarm. When developing prosthetic devices, basing decisions on incorrect results (i.e. not including prior information in the structural modeling of human factors) increases the likelihood of reduced satisfaction with the product or even harm to the user. Therefore, one conclusion of this paper is that switching to probabilistic inference when it comes to research in the field of prosthetics is a promising approach to gather clearer information about the perception and behavior of prosthesis users. Whether our informed model provides better results than a competing uninformed model or the frequentist approach will need to be determined.

### Model design and limitations

Using informed prior distributions is one point of criticism of Bayesian inference. Could the priors chosen be ill-informed? How much impact does an informed prior, regardless of its objective applicability, have on the outcome? In the case of this paper, the parameter distributions were informed by using prior knowledge that was not directly gathered from these parameters, i.e. through pilot studies involving the exact upcoming questionnaire. Consequently, every bit of information gathered through the preliminary study should be examined cautiously. The item-factor classifications and factor loading priors appear to be the most objective source of prior knowledge since expert studies are a common technique when it comes to confirmatory statistical models. To correct for possible biases in said extraction, the resulting model will be tested against a competing, uninformed model to investigate the validity of the chosen prior distributions. If the data do not provide enough likelihood to overcome uninformed prior distributions in a noticeable way (i.e. parameters different from zero remain credibly different from zero, no matter the prior), then adjustments to the model have to be considered. Additionally, there are still 147 uninformed prior distributions on parameters in the model. These uninformed parameters might, even though it is not likely, overshadow the statistical power of the informed parameters rendering the differentiation between informed and uninformed models trivial.

### Future research

The next step of validating the proposed model is to gather empirical data and check the assumptions made in the parameter prior distributions for validity. This will include a comparison with a competing model in which the informed distributions described here will be left uninformed. In order to gather a wider array of subjects, a cross-validated translation of the currently German set of items will be compiled. Furthermore, an identification of the structural model is still required as this study makes no assumption about the regression parameters between resulting latent factors. Once evaluated, the model can be enhanced to respect different user stereotypes, granting individual parameter values to participants with different medical sources of amputation or demographic factors. Special interest also lies in the question of if and how body schema integration can be achieved through psychological interventions or technological improvements or how expert knowledge acquisition can improve socket fit, both factors being proposed important influences on overall satisfaction.
